# Proteome profiling of hippocampus reveals the neuroprotective effect of mild hypothermia on global cerebral ischemia–reperfusion injury in rats

**DOI:** 10.1038/s41598-023-41766-2

**Published:** 2023-09-02

**Authors:** Jiajia Wang, Xiaopeng Sun, Yuting Dai, Yuan Ma, Mingshan Wang, Xiaoxia Li, Weiwei Qin

**Affiliations:** 1 Department of Anesthesiology, Qingdao Hospital, University of Health and Rehabilitation Sciences (Qingdao Municipal Hospital), Qingdao, 266071 China; 2 Department of Anesthesiology, Qingdao Municipal Hospital, Qingdao University, Qingdao, 266071 China; 3 Department of Anesthesiology, Qingdao Hiser Hospital Affiliated of Qingdao University (Qingdao Traditional Chinese Medicine Hospital), Qingdao, 266000 China; 4 Department of Genetics and Cell Biology, Basic Medical College, Qingdao University, Qingdao, 266071 China

**Keywords:** Stroke, Proteomics

## Abstract

Cerebral ischemia is one of the leading causes of disability and mortality worldwide. Blood reperfusion of ischemic cerebral tissue may cause cerebral ischemia–reperfusion (IR) injury. In this study, a rat model of global cerebral I/R injury was established via Pulsinelli’s four-vessel occlusion (4-VO) method. The liquid chromatography-tandem mass spectrometry (LC–MS/MS) and bioinformatics analysis were employed to examine the ipsilateral hippocampus proteome profiles of rats with/without MH (32 °C) treatment after IR injury. Totally 2 122 proteins were identified, among which 153 proteins were significantly changed associated with MH (n = 7 per group, fold change-1.5, *p* < 0.05). GO annotation of the differentially expressed proteins (DEPs) revealed that cellular oxidant detoxification, response to zinc ions, aging, oxygen transport, negative regulation of catalytic activity, response to hypoxia, regulation of protein phosphorylation, and cellular response to vascular endothelial growth factor stimulus were significantly enriched with MH treatment. The KEGG analysis indicated that metabolic pathways, thermogenesis, pathways of neurodegeneration, chemical carcinogenesis—reactive oxygen species, fluid shear stress and atherosclerosis, and protein processing in endoplasmic reticulum were significantly enriched with MH treatment. Importantly, changes in 16 DEPs were reversed by MH treatment. Among them, VCAM-1, S100A8, CaMKK2 and MKK7 were verified as potential markers associated with MH neuroprotection by Western blot analysis. This study is one of the first to investigate the neuroprotective effects of MH on the hippocampal proteome of experimental models of cerebral IR injury. These DEPs may be involved in the most fundamental molecular mechanisms of MH neuroprotection, and provide a scientific foundation for further promotion of reparative strategies in cerebral IR injury.

## Introduction

Cerebral ischemia contributes significantly to morbidity and mortality in a wide range of pathologies, including stroke, trauma, cardiac arrest and perinatal hypoxic-ischemic encephalopathy. Current treatment options for cerebral ischemia are limited, and thrombolytic therapy is the most effective evidence-based treatment^1^. Restoration of blood supply by reperfusion can salvage ischemic tissue but reperfusion per se causes tissue injury, which is called cerebral ischemia–reperfusion (IR) injury^2,3^. As endovascular therapy for ischemic stroke has advanced in recent years, the challenge of devising neuroprotective strategies for cerebral IR injury has grown. However, the existing treatment for cerebral IR injury includes supportive therapy, symptomatic treatment, targeted temperature management (TTM) and hyperbaric oxygen therapy, and there is currently no more effective treatment. TTM is one of the most robust neuroprotectants studied to date.

Growing evidence suggests that mild hypothermia (MH) adjuvant therapy has been widely used in clinical treatment of anoxic brain diseases^4^. MH improves reperfusion injury and its poor prognosis in ischemic stroke and plays a neuroprotective role^5^. It was previously reported that MH influences multiple aspects of brain physiology in the acute, sub-acute and chronic phases of cerebral ischemia. MH may affect pathways leading to excitotoxicity, inflammation and free radical production, as well as blood flow, metabolism and blood–brain barrier (BBB) integrity^6^. It may also influence neurogenesis, gliogenesis and angiogenesis after injury. However, the exact mechanism by which mild hypothermia improves brain injury after IR has not been determined. It is likely that no single factor can explain the neuroprotection effect, but understanding its myriad effects may shed light on the neuroprotective mechanisms of MH.

Mass spectrometry (MS)-based proteomics is a powerful tool that provides assessments with thousands of proteins investigated simultaneously. Recently, a number of proteome-wide studies have explored molecular expression profiles after different types of cerebral ischemia. Hundreds of differentially expressed proteins involved in multiple bioprocesses were identified in the cortex^7^, hippocampus^8^, plasma^9^, and subventricular zone^10,11^. These findings provide an ample reserve of therapeutic targets and/or biomarker candidates for subsequent research. Because most injury mechanisms are sensitive to temperature, the molecular events occurring with hypothermia treatment are needed. Using a 4-labeled iTRAQ proteomics approach, Cheng et al. found that the expression of four candidate molecules (plasminogen, antithrombin III, fibrinogen gamma chain, transthyretin) was significantly suppressed by hypothermia after traumatic brain injury (TBI)^12^. Although the expressions of certain proteins were studied after cerebral IR injury, both the differentially expressed proteome profiles and the network of neuroprotective mechanisms of MH at the proteome level need to be further explored.

In the present study, we established a rat model of global cerebral I/R injury via Pulsinelli’s four-vessel occlusion (4-VO) method. Then label free LC–MS/MS and bioinformatics analysis were applied to determine the changes in differentially expressed hippocampal proteins associated with MH treatment. We intend to elucidate the possible role of MH in the molecular pathways following cerebral IR injury.

## Methods

### Animal experiments

Male Wistar rats (weight, 280–320 g) were purchased from Charles River China (Beijing, China). All animals were maintained on a standard laboratory diet with a controlled indoor temperature (21 ± 2 °C), humidity (65–70%) and 12/12 h light–dark cycle conditions. All methods were carried out in accordance with relevant guidelines and regulations of the National Health Commission and the Ministry of Science and Technology and conformed to the guidelines for animal research. The experiments were reviewed and approved by the Qingdao Municipal Hospital Medical Ethics Committee (2021Y39).

Cerebral ischemia was established by Pulsinelli’s four-vessel occlusion (4-VO) method as previously described^13^. Forty-two rats were randomly divided into three groups: sham-operation group, normothermia cerebral IR group, and therapeutic hypothermia cerebral I/R group (14 rats per group). Animals were anesthetized with 1% pentobarbital sodium (40 mg/kg body weight). For rats in the I/R group, the bilateral pterygoid foramens were surgically exposed and the bilateral vertebral arteries were electrocauterized, and the bilateral common carotid arteries were surgically exposed. Rats were allowed to recover for 24 h. Then ischemia was induced by occluding the common arteries with artery clamps for 15 min. Rats lost righting reflex within 30 s and pupils were dilated and unresponsive to light were selected for the later experiments. For rats in the sham-operation group, vessels were exposed but without occlusion.

Mild hypothermia (32 ± 0.5 °C) was induced from the onset of ischemia and maintained for 4 h. Core body temperature was continuously monitored with temporalis muscle and rectal temperature probes. Mild hypothermia was induced by applying an ice blanket over the dorsum of prone rats until a body temperature of 32 ± 0.5 °C was achieved. After 4 h of hypothermia, the rats were allowed to gradually rewarm back to their baseline temperature (37 ± 0.5 °C) during a 1 h period using heating lamps. The hippocampus tissues were collected at 24 h after IR injury, and then immediately stored at − 80 °C for later analysis.

Morris water maze was conducted using a water maze (ZS-Morris, Beijing). The diameter of the water tank was 160 cm. Each rat received four-trials per-day training in the water maze, and lasted for four days. The maximum time to search for hidden platforms was 60 s. Once the rat found the submerged platform, it was allowed to stay on it for 10 s, the time spent to reach the platform (the escape latency) and path length were recorded. Spatial probe test was conducted on the fifth day, the platform was removed and the rats swam freely for 60 s. The time and distance that rats spent in the target quadrant were recorded.

### Histological analysis

For histopathology, animals were anesthetized 24 h after the IR injury, and then cardiac regions were exposed by thoracotomy. Left ventricular cannulation of the ascending aorta was performed. Rats were then perfused with 200 mL saline solution to flush the blood followed by 200 ml of 4% paraformaldehyde (pH 7.2–7.4) for perfusion fixation. Following complete perfusion, whole brains were harvested. Whole-brain tissues were fixed in fresh 4% paraformaldehyde at 4 °C. The fixed brain tissues were embedded in paraffin, and then sectioned (4 μm) and stained with hematoxylin and eosin (HE), and Nissl staining to reveal histopathological lesions.

### Hippocampus sample preparation

The ipsilateral hippocampus tissues were pulverized to a fine powder on dry ice, which was then suspended in lysis buffer (300 mL), containing protease inhibitors. Tissue lysis continued for 60 min at 4 °C, insoluble material was removed by centrifugation at 14 000 g for 10 min. The protein concentration was measured using a BCA Protein Assay kit (Thermo Fisher, USA). Trypsin (Promega, USA) was used for protein digestion, via filter-aided sample preparation methods^14^. Briefly, 50 µg of the protein sample was loaded onto a 10-kDa filter unit (Pall, USA). DTT (4.5 mM) was added to the protein solution for 1 h at 37 °C. Indoleacetic acid was added (10 mM) for 30 min at room temperature in the dark. The proteins were digested with trypsin (enzyme-to-protein ratio of 1:50) for 14 h at 37 °C. The peptide samples were lyophilized for LC–MS/MS analysis.

### LC–MS/MS analysis

An Orbitrap Fusion Lumos Tribrid mass spectrometer coupled with an EASY-nLC 1000 HPLC system (Thermo Scientific, Germany) was used to analyze the peptide sample, as previously described^15,16^. In detail, the digested peptides were loaded onto a trap column (75 µm × 2 cm, 3 µm, C18, 100 A°), and then transferred to a reversed-phase analytical column (50 µm × 250 mm, 2 µm, C18, 100 A°). The elution acetonitrile gradient was set from 5 to 30% (350 nL/min) for 90 min. The MS parameters were set as follows: the full scan at 120 000 from 350 to 1 550 m/z, the cycle time (3 s) was set to top speed mode, the AGC was set to 1E6, and the maximum injection time was set to 100 ms. MS2 scans were acquired with an isolation window of 2 Da at a resolution of 30 000, HCD was set to 32%; the AGC target was set to 5E5 and the maximum injection time was set to 50 ms.

### Data processing

The raw data files were processed using Progenesis QIP and Mascot software, as previously described^16^. Briefly, the search parameters were set as follows: SwissProt *Rattus* database (containing 8086 sequences), trypsin digestion, parent ion mass tolerance of 10 ppm, fragment ion mass tolerance of 0.02 Da, fixed modification of carbamidomethylated cysteine (+ 58.00 Da); variable modifications of deamidated glutamine asparagine (+ 0.984 Da) and oxidized methionine (+ 15.995 Da). The default parameters were used for other settings. After normalization, the mass spectrometry peak intensity was used to analyze differential proteins between groups. The false discovery rate (FDR) was set to 0.01 at the protein level. The differential proteins were selected using one-way ANOVA. Significance was set at a fold change of 1.5 and a* p* value < 0.05.

### Bioinformatics analysis

The Database for Annotation, Visualization and Integrated Discovery (DAVID) 6.8 (https://david.ncifcrf.gov/) was used to analyze the functional annotation of the differential proteins^17,18^. In this study, significant GO enrichment was defined as *P* < 0.05. Protein–protein interaction networks were constructed via the STRING database (http://www.string-db.org), which is a database of known and predicted protein interactions, including direct (physical) and indirect (functional) associations.

The 'Wu Kong' platform (https://www.omicsolution.org/wkomics/main/) was used for statistical analysis.

### Western Blot analysis

Hippocampa; proteins (30 μg) were separated by 12% SDS-PAGE, and the gel was cut according to the mass marker. Then the targeted proteins were transferred to 0.45 nm polyvinylidene difluoride (PVDF) membranes. After blocking in TBST buffer (1X TBS containing 0.1% Tween 20) with 5% (w/v) skimmed milk for 40 min at room temperature, the membranes were incubated with diluted primary antibodies (anti-VCAM-1 antibody; anti-S100A8 + S100A9 antibody; anti-CaMKK2 antibody; anti-MKK7 antibody; Abcam, China) at a dilution of 1:1000 with gentle shaking overnight at 4 °C. After washing 3 times with TBST buffer, the membrane was probed with secondary antibodies (dilution 1:5000) coupled to horseradish peroxidase at room temperature for 1 h. Densitometry analysis was performed using ImageJ software, and protein-to-β-actin ratios were expressed as the means ± SD from four independent experiments. *P* values were calculated using one-way ANOVA.

### Ethics approval and consent to participate

Male Wistar rats (280–300 g) were purchased from Charles River China (Beijing, China). The animal experiments were reviewed and approved by the Qingdao Municipal Hospital Medical Ethics Committee (2021Y39).

### Statement on arrive guidelines

We declared that this study was carried out in compliance with the ARRIVE guidelines.

## Results and discussion

### Mild hypothermia attenuates neurological damage and cognitive dysfunction

**Figure 1:**
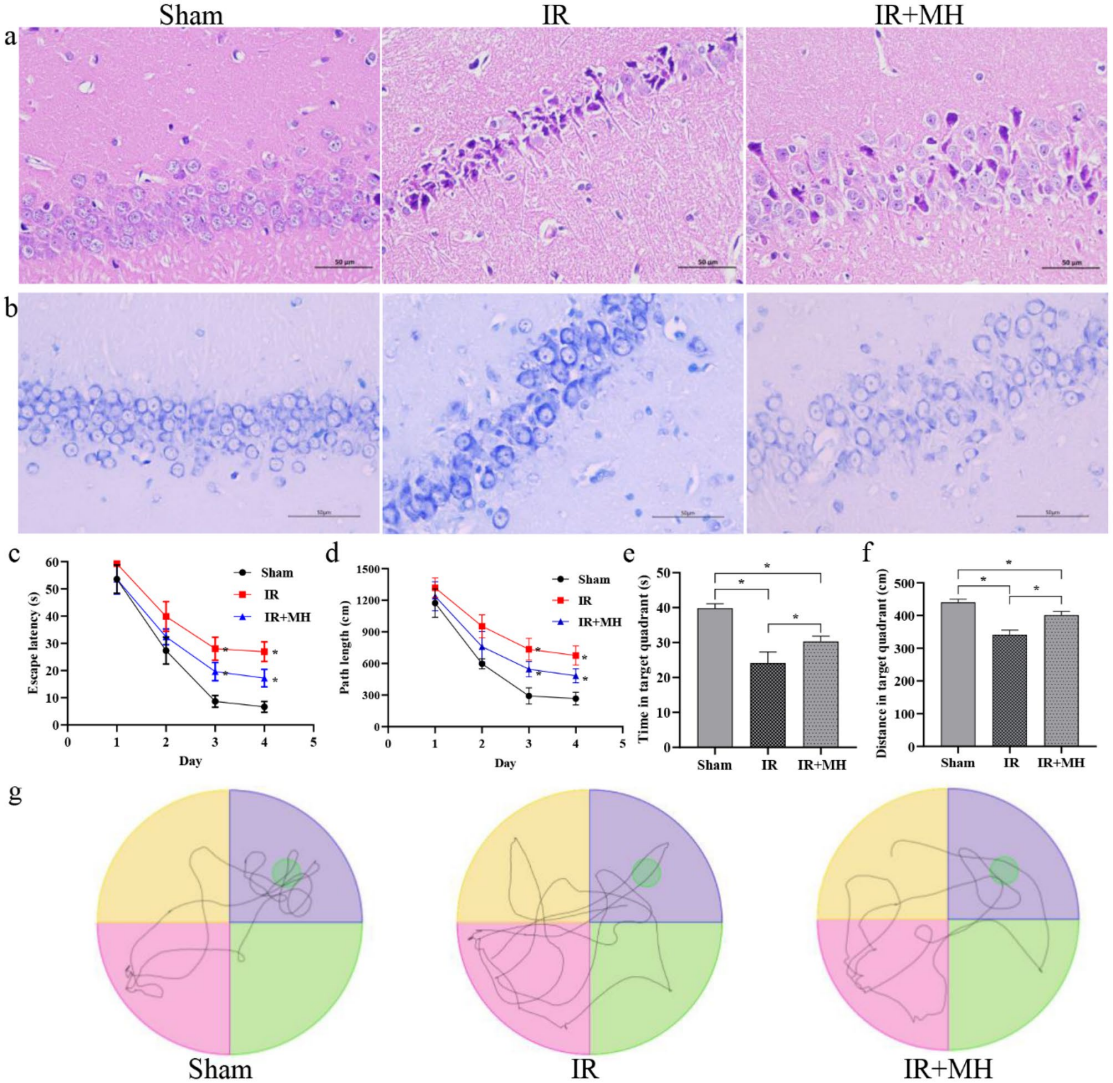
Protective effects of mild hypothermia on hippocampus neurological damage and cognitive dysfunction after cerebral ischemia-perfusion injury. (**a**) HE staining; (**b**) Nissl staining; the time (**c**) and path length (**d**) that rats spent to find the platform during 4 training days; The time (**e**) and distance (**f**) that rats spent in the target quadrant in the probe test; (**g**) the routes of rats in the probe test. Sham: Sham-operation group; IR: cerebral IR injury followed by normothermia (37 °C) group; IR + MH: cerebral IR injury followed by 4 h of MH (32 °C) group. Scale bars = 50 µm. Results are presented as the mean ± standard deviation (SD). *P < 0.05.

To examine ischemia induced histological damage and the neuroprotective effect of MH, HE and Nissl staining were conducted on brain sections of hippocampal CA1 region, which is selectively vulnerable following IR injury. HE staining revealed that no neuron morphology abnormalities were observed in the sham group; the number of neurons was reduced and typical apoptotic cells (shrunken cell bodies and nuclear pyknosis) were observed in IR group; and MH markedly ameliorated pathological changes in IR + MH group compared to the IR group (Figs. 1a, S1). Nissl staining showed intact pyramidal neurons, abundant cytoplasm and Nissl bodies of the hippocampus in sham group; cell death characteristics (loosely arranged neurons and fuzzy cells outlines, loss and light color staining) exhibited at 24 h after reperfusion in IR group; neuronal damages in IR + MH group was significantly reduced compared to the IR group (Fig. 1b).

The escape latency and path length of all 3 groups decreased progressively during the 4 training days (Fig. 1c, d). The time that rats spent in the target quadrant and the swimming distance were all analyzed to determine the differentials of the spatial memory ability. As shown in Fig. 1e, f, compared to the sham group, the time and swimming distance of the IR and IR + MH groups were shorter (*P* < 0.05). With MH treatment, the time and swimming distance of the IR + MH group were longer than those of the IR group (*P* < 0.05). The representative pathways of rats from the 3 groups in the probe test are shown in Fig. 1g. Rats from the IR group could hardly locate the target quadrant correctly. The data indicated that there was memory impairment in IR rats, and MH treatment could improve the memory ability in IR rats.

### Profiling of hippocampus proteome after cerebral IR injury with/without MH

Although MH has long been associated with cerebral IR injury, its specific role in mediating neuroprotective signaling pathways is not well understood. Using a quantitative proteomics protocol, we performed global profiling of protein expression from the ipsilateral hippocampus tissue of rats (n = 7 per group), which were subjected to sham or 4-VO operation, followed by 4 h of MH or normothermia.

**Figure 2:**
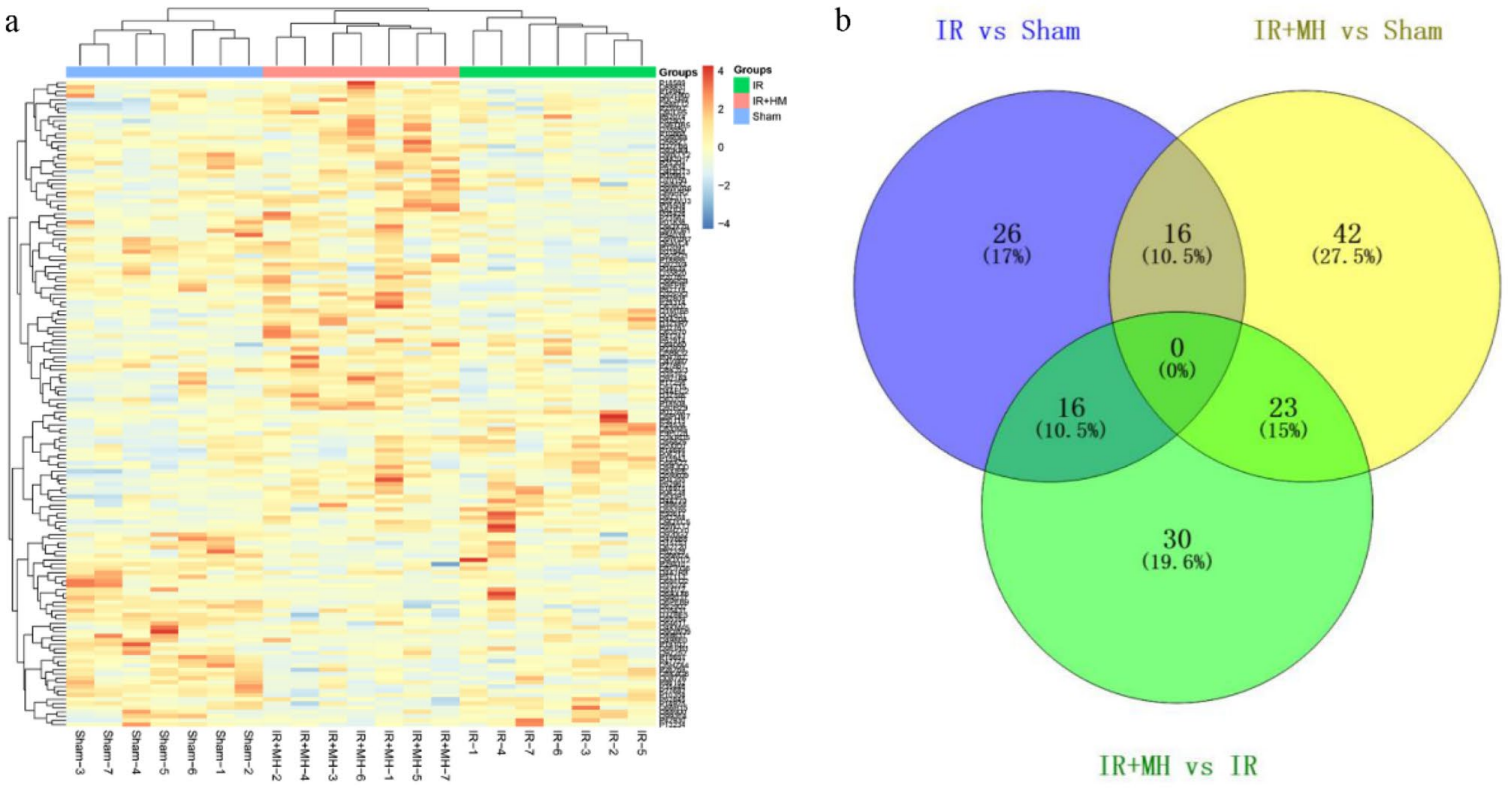
The differentially expressed proteins in hippocampus under cerebral IR injury followed by normothermia or MH. (**a**) Hierarchical cluster analysis; (**b**) the Venn diagram. Sham: Sham-operation group; IR: cerebral IR injury followed by normothermia (37 °C) group; IR + MH: cerebral IR injury followed by 4 h of MH (32 °C) group.

After LC–MS/MS analysis, 2 122 proteins were identified with at least 1 unique peptide (FDR < 1%). Among these, 153 proteins were significantly changed (fold change-1.5, *p* < 0.05). The quantification of differentially expressed proteins (DEPs) is shown in Table S1. The results of hierarchical cluster analysis for DEPs showed that MH could significantly reverse many protein expression trends compared with the IR group (Fig. 2a). After cerebral IR injury, 58 proteins were changed compared to sham controls, with 26 proteins being upregulated and 32 proteins being downregulated; With MH treatment, 81 proteins were changed compared to sham controls, with 46 proteins being upregulated and 35 proteins being downregulated; MH changed 69 proteins after cerebral IR injury, with 51 proteins being upregulated and 18 being downregulated. The overlap of the DEPs identified between different groups is shown as a Venn diagram (Fig. 2b). Interestingly, we noticed that the expression trends of 16 DEPs were reversed by MH (Fig. 2b). Four DEPs were highly expressed in the cerebral IR injury rats and significantly reversed by MH treatment, while the expression of the other 12 DEPs displayed low expression in the IR injury rats and then high expression with MH treatment. The details of the 16 DEPs are listed in Table 1.

**Table 1:** The differentially expressed proteins in hippocampus were closely associated with mild hypothermia.

Uniprot ID	Protein name	Human ortholog	IR versus Sham		IR + MH versus IR	
			FC	*p* value	FC	*p* value
P02764	Alpha-1-acid glycoprotein	P02763	2.99	0.00	− 3.16	0.00
P29534	Vascular cell adhesion protein 1	P19320	2.74	0.00	− 1.70	0.04
P50115	S100 calcium binding protein A8	P05109	2.22	0.03	− 2.43	0.02
P42930	Heat shock protein beta-1	P04792	1.92	0.03	− 1.66	0.05
Q5XIM5	Protein CDV3 homolog	Q9UKY7	1.86	0.04	− 1.85	0.03
P04639	Apolipoprotein A-I	P02647	− 1.52	0.02	1.64	0.03
Q5FWU3	Autophagy-related protein 9A	Q7Z3C6	− 1.52	0.01	2.16	0.00
Q66HR2	Microtubule-associated protein RP/EB family member 1	Q15691	− 1.55	0.00	1.58	0.01
Q9R064	Golgi reassembly-stacking protein 2	Q9H8Y8	− 1.56	0.01	1.71	0.03
O88831	Calcium/calmodulin-dependent protein kinase kinase 2	Q96RR4	− 1.70	0.05	2.37	0.01
Q9WTY2	GTP-binding protein REM 2	Q8IYK8	− 1.74	0.05	1.59	0.05
Q925G1	Hepatoma-derived growth factor-related protein 2	Q7Z4V5	− 1.79	0.03	1.94	0.03
P18886	Carnitine O-palmitoyltransferase 2, mitochondrial	P23786	− 1.84	0.00	1.82	0.03
D4A1R8	Copine-1	Q99829	− 2.41	0.01	1.98	0.04
P62634	Cellular nucleic acid-binding protein	P62633	− 2.89	0.04	3.71	0.01
Q4KSH7	Dual specificity mitogen-activated protein kinase kinase 7	O14733	− 5.04	0.01	4.80	0.02

### Function annotation and KEGG pathway analysis of DEPs associated with MH

**Figure 3:**
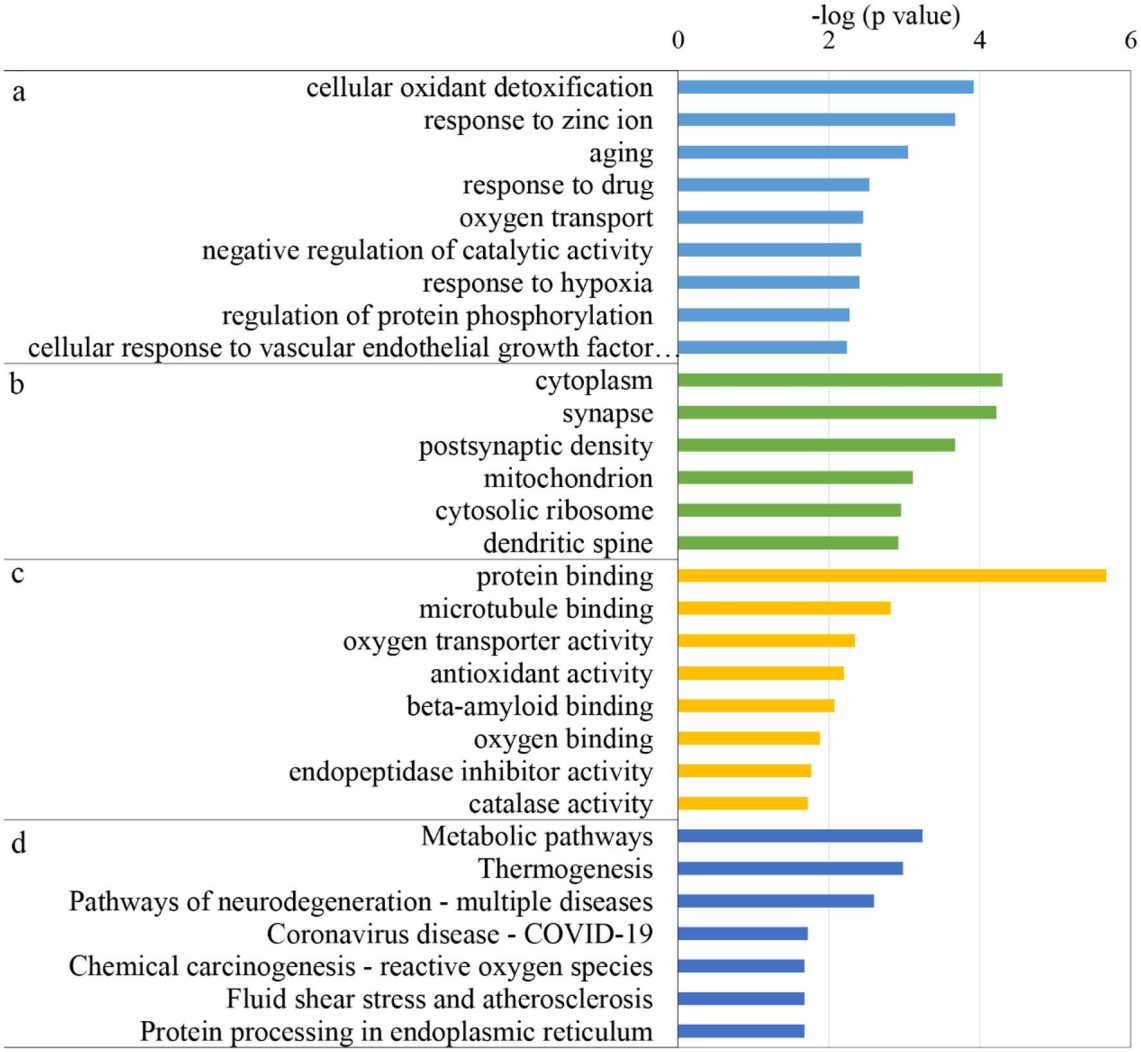
GO and KEGG enrichment analysis of the DEPs associated with mild hypothermia. (**a**) Biological process; (**b**) cellular component; (**c**) molecular function; (**d**) KEGG pathway (*p* value < 0.05).

The functional annotation of the 153 DEPs consisted of sorting them into the “biological process”, “cellular component” and “molecular function” categories using DAVID. In the biological process category, cellular oxidant detoxification, response to zinc ion, aging, oxygen transport, negative regulation of catalytic activity, response to hypoxia, regulation of protein phosphorylation, and cellular response to vascular endothelial growth factor stimulus were overrepresented with MH treatment (Fig. 3a). In the cellular component category, most of these differentially expressed proteins were associated with cytoplasm, synapse, postsynaptic density, and mitochondrion (Fig. 3b). In the molecular function category, protein binding, microtubule binding, oxygen transporter activity, antioxidant activity, beta-amyloid binding, and oxygen binding were overrepresented with MH treatment (Fig. 3c). MH treatment might regulate the expression of related molecules of cellular function and maintenance function, including vascular cell adhesion molecule 1 (VCAM-1), S100 calcium binding protein A8 (S100A8), crystallin alpha B (Cryab), CREB-regulated transcription coactivator 1 (Crtc1), heat shock protein beta-1 (Hspb1), autophagy-related protein 9A (Ata9a), and calcium/calmodulin-dependent protein kinase kinase 2 (CaMKK2), and these proteins may be involved in the neuroprotective function.

We examined which molecular pathways these DEPs may be involved in using KEGG pathway analysis^19,20^. Seven pathways were selected with the most significant enrichment (*p* value < 0.05) by KEGG analysis (Fig. 3d). In particular, metabolic pathways, thermogenesis, pathways of neurodegeneration—multiple diseases, chemical carcinogenesis—reactive oxygen species, fluid shear stress and atherosclerosis, and protein processing in endoplasmic reticulum were enriched, which may be closely related to the actual in vivo changes in the hippocampus after cerebral IR injury. Therefore, the proteins of these common pathways were used for the next analysis.

### Protein–protein interactions of DEPs associated with MH

**Figure 4:**
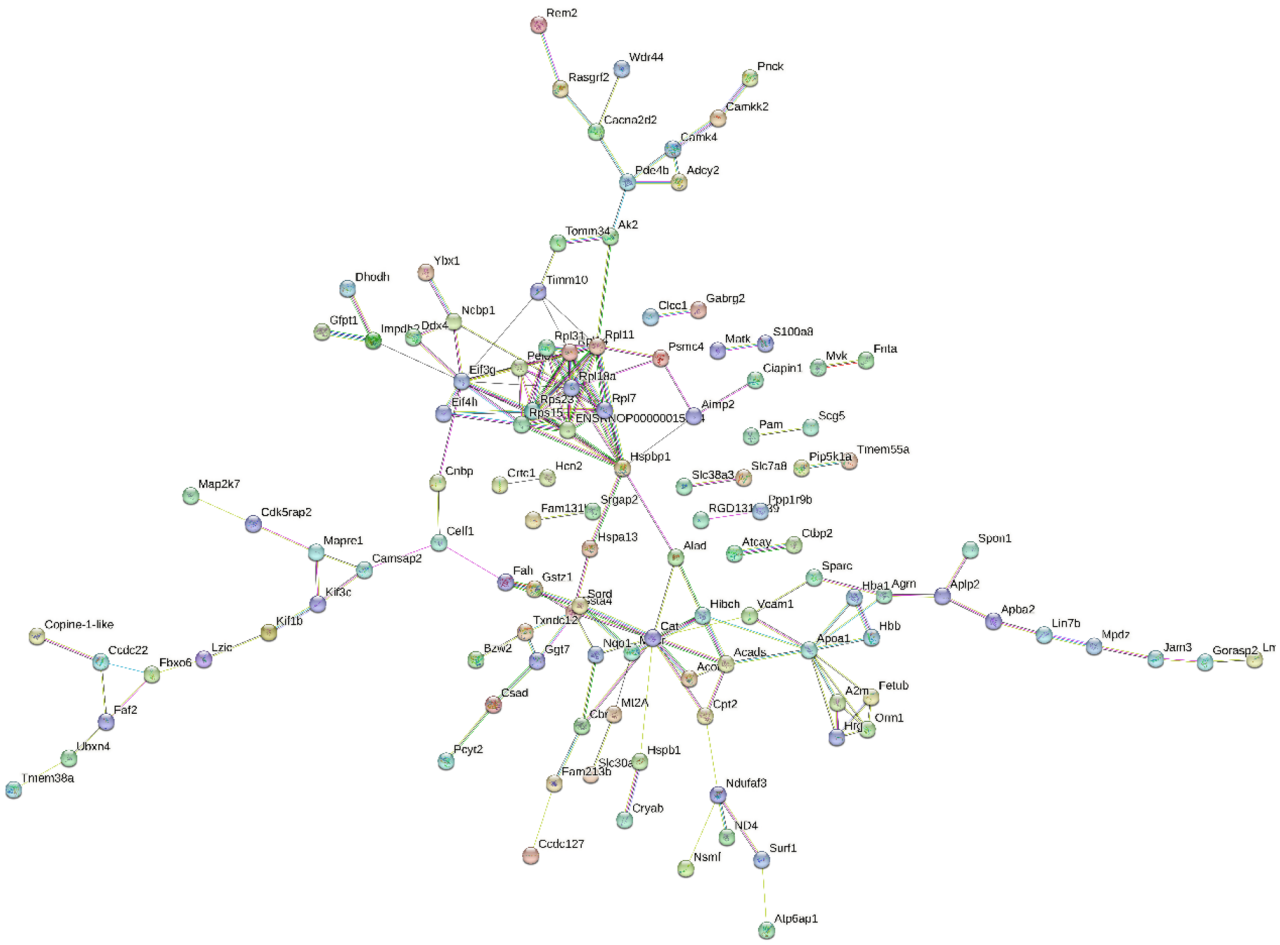
STRING PPI network analysis of the DEPs associated with mild hypothermia. The number of nodes is 150, the average node degree is 2.36, and the average local clustering coefficient is 0.432 (*p* value < 0.01).

To better understand the neuroprotective mechanisms of MH, a protein–protein interaction (PPI) network for 153 DEPs was constructed using STRING (Fig. 4). The STRING PPI network analysis showed that the average node degree was 2.36, the average local clustering coefficient was 0.432, and the PPI enrichment *p* value was 0.0003. The above results revealed that these DEPs had more interactions among themselves than would be expected for a random set of proteins of similar size. Such an enrichment pattern indicates that apolipoprotein A1 (Apoa1), vascular cell adhesion protein 1 (VCAM-1), catalase (Cat), 26S proteasome regulatory subunit 6B (Psmc4) may play a key modulatory role in neuroprotective effects under MH.

### Validation of DEPs associated with MH by Western blotting

According to the bioinformatic analyses of the DEPs, several representative proteins were selected for further validation by western blotting in extended samples. The selected DEPs closely associated with MH in cerebral IR injury included vascular cell adhesion protein 1 (VCAM-1), S100 calcium binding protein A8 (S100A8), calcium/calmodulin-dependent protein kinase kinase 2 (CaMKK2), and dual specificity mitogen-activated protein kinase kinase 7 (MKK7), which are included in significantly enriched GO terms and protein–protein interaction network nodes (Figs. 3, 4).

**Figure 5:**
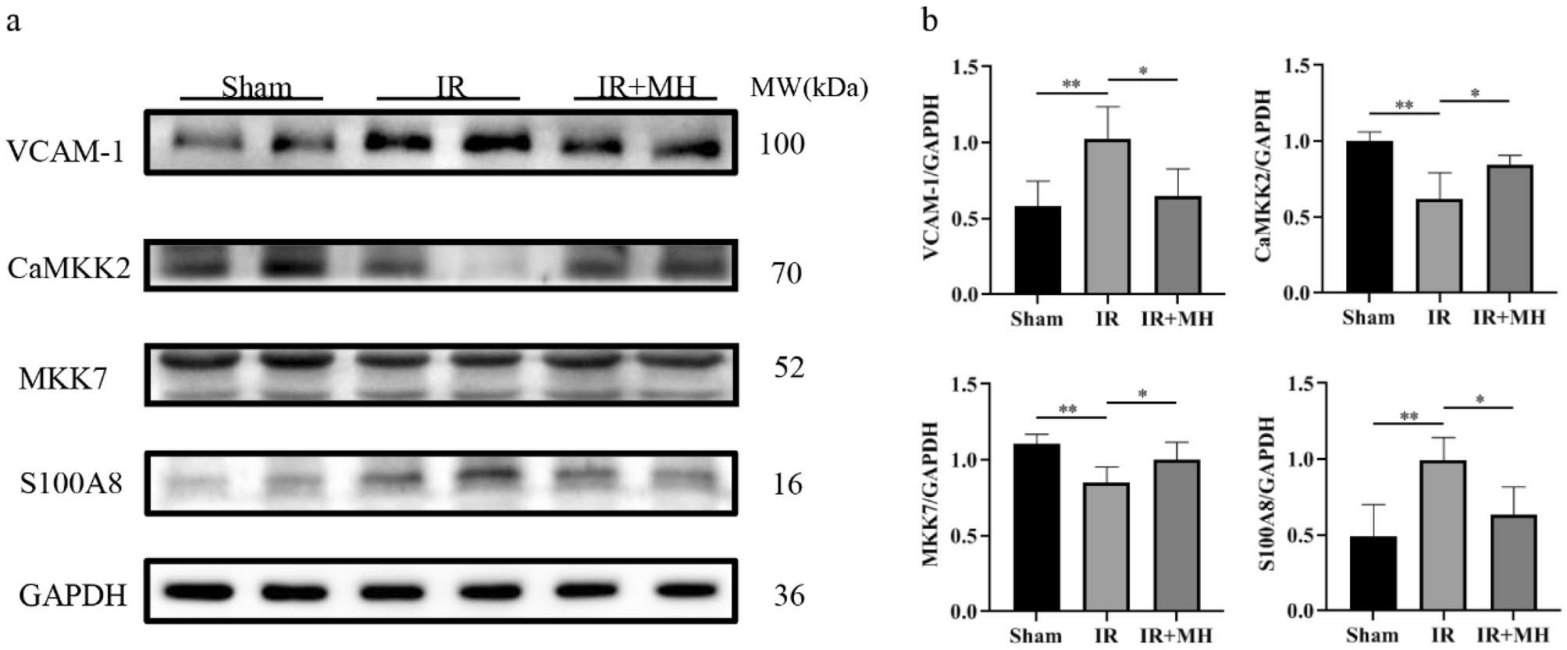
Validation of DEPs associated with mild hypothermia by Western blotting. (**a**) Representative blots visualizing the levels of VCAM-1, CaMKK2, MKK7 and S100A8 in the three groups. GAPDH was used as the loading control. (**b**) Densitometric values are expressed as the mean ± SD normalized to GAPDH (n = 4 per group). Sham: Sham-operation group; IR: cerebral IR injury followed by normothermia (37 °C) group; IR + MH: cerebral IR injury followed by 4 h of MH (32 °C) group. *p < 0.01; **p < 0.001.

The contents of the selected DEPs in the ipsilateral hippocampus samples were calculated and are shown in Fig. 4. As expected, the WB results of the four DEPs were consistent with the results of the proteomic analysis. Significantly different amounts of VCAM-1 (*p* < 0.01), S100A8 (*p* < 0.01), CaMKK2 (*p* < 0.01), and MKK7 (*p* < 0.001) were found after cerebral IR injury with/without MH treatment (Figs. 5, S2). VCAM-1 and S100A8 were highly expressed in cerebral IR injury rats and significantly reversed by MH treatment. The expression of CaMKK2 and MKK7 was lower in the IR injury rats and then elevated to basal levels with MH treatment.

## Discussion

In this study, we aimed to explore the effect of mild hypothermia (MH) on protein expression in the hippocampus and the molecular mechanism of hypothermic neuroprotection after cerebral IR injury. Overall, for the first time we systematically investigated dynamic changes in hippocampus proteome in a 4-VO rat model with/without MH based on proteomics analysis. A total of 2 122 proteins were identified (FDR < 1%), of which 153 proteins were differentially expressed associated with MH treatment. Many of these differentially expressed proteins (DEPs) were involved in reactive oxygen species, endoplasmic reticulum function, and vascular endothelial growth. Importantly, changes in 16 DEPs were reversed by MH treatment in the cerebral ischemia, and all these DEPs have counter parts in human (Table 1). After WB validation, the expression of VCAM-1, S100A8, CaMKK2 and MKK7 were confirmed to be consistent with the results of the proteomic analysis. These results suggest that human hippocampus proteome may change to some extent under MH treatment, and provide insight into the potential mechanisms of MH neuroprotective effect.

Vascular endothelial cells and leukocytes express several inflammatory cell adhesion molecules (CAMs), which involved in the pathogenesis of ischemic cerebrovascular diseases^21^. VCAM-1 expression on endothelial cells is induced by TNF- and IL-1, and plays an important role in leukocyte firm adhesion and transmigration. Upregulation of soluble VCAM-1 has been demonstrated in patients with ischemic stroke, although evidence is contradictory. The downregulation of VCAM-1 improved stroke outcomes in pre-clinical models. In a middle cerebral artery occlusion (MCAO) mice model, VCAM-1 gene silencing using small interfering RNA effectively reduced the infarct volume and attenuated leukocyte infiltration and cerebral IFN-g levels^22^. However, intravenous injection of anti-VCAM-1 antibody failed to show a neuroprotective effect either in rats or in mice^23^. In the current study, we detected a significant up-regulation of VCAM-1 in the hippocampus of cerebral IR injury rats, which was then reversed by MH treatment, indicating that MH may play a neuroprotective role by reducing the expression of VCAM-1.

S100A8 is a calcium- and zinc-binding protein which plays a prominent role in the regulation of inflammatory processes and immune response. Predominantly found as calprotectin (S100A8/a9) which has a wide plethora of intra- and extracellular functions. In the central nervous system (CNS), S100A8/a9 is abundantly expressed in microvascular endothelial cells and microglial cells^24,25^, and involved in the pathology of various cerebral diseases, including Alzheimer’s disease^26^, cerebral ischemia^27^. The expression of S100A8 was markedly upregulated in oxygen–glucose deprivation and reoxygenation (OGD/R)-induced BV2 microglia cell injury, and silencing S100A8 could alleviate inflammation, oxidative stress and the apoptosis by upregulating GAB1 expression^28^. Based on proteomics analysis, the level of S100A8 increased 9.5-fold in cerebrospinal fluid (CSF) after global hypoxia–ischemia in newborn piglets^9^. Recently, scientists form China reported that increased plasma S100A8/A9 concentrations were significantly associated with poor prognosis at 3 months after ischemic stroke in 2 separate cohorts and pooled analysis of 4 785 patients^29^. Similarly, Marta-Enguita et al. from Italy found that plasma S100A8/A9 is an independent predictor of 3-month mortality after acute ischemic stroke^30^. In the current study, the content of S100A8 in the hippocampus of IR injury group increased, and then was reversed by MH treatment, indicating that MH may play a neuroprotective role by reducing the inflammatory response by inhibiting the expression of S100A8.

CaMKK is a serine/threonine-specific protein kinase, and could be directly activated by rising intracellular calcium. This kinase has 2 isoforms α and β, both of which are expressed in the nervous system and hematopoietic cells^31^. Compelling evidence has suggested that CaMKK is critical in neuronal survival when cells are under ischemic stress. McCullough et al. firstly found inhibition of CaMKK2 either pharmacologically or genetically was detrimental in cerebral ischemia mice model^32^. Conversely, overexpression of CaMKK2 reduced brain injury in cerebral ischemia aged mice^33^. Furthermore, using pharmacological and gene knockdown approaches, Sun et al. demonstrated that CaMKK2 inhibition reduced endothelial cell viability, exacerbated inflammatory responses and aggravated blood–brain barrier (BBB) impairment in vitro and in vivo^34^. In the current study, we detected a significant decrease of CaMKK2 in cerebral IR injury rats, and then increased about twofold after MH treatment, suggesting that CaMKK2 may be a key endogenous protective molecule in MH neuroprotection.

Dual specificity protein kinase (MKK), an essential component of the MAP kinase signal pathway, is the only known kinase to directly activate the stress-activated protein kinase/c-Jun N-terminal kinases (JNKs) by phosphorylation. MKK4/7 is important to trigger JNK activity, and has a specific role in JNK signal transduction pathway activated by pro-inflammatory cytokines. In mouse brain, deletion of MKK4/7 causes developmental defects, indicating that MKK-JNK signaling is necessary not only for stress responses but also for early development of the brain^35^. However, the role of MKK4/7 in cerebral ischemia injury are still controversial. Pan et al. reported that MKK7 was rapidly activated in transient brain ischemia in the CA1 hippocampal region at 30 min and 1 day of reperfusion, while the protein levels of MKK7 showed no changes after reperfusion^36^. Repici et al. reported that MKK7 did not show any significant increase of phosphorylation in either core or penumbra after MCAO, and the protein levels of MKK7 decreased at 30 min, 1 h and 3 h after ischemia^37^. Vercelli et al. reported that specific inhibition of MKK7 was sufficient to prevent excitotoxicity in vitro as well as in vivo in two models of cerebral ischemia, obtained by electrocoagulation and by thromboembolic occlusion of the middle cerebral artery^38^.

In the current study, the hippocampus MKK7 content decreased nearly five-fold, with MH treatment the content elevated to basal level, indicating that MKK7 may play an important role in MH neuroprotection. Thus, further studies are needed to fully elucidate how p-MKK7/MKK7 supports neural functions in MH.

Above all, the present study is a preliminary proteomic study of neuroprotection of MH in cerebral IR injury, and suggesting that VCAM-1, S100A8, CamKK2 and MKK7 may be involved in the most fundamental molecular mechanisms of MH neuroprotection. In future studies, further investigations are needed to precisely understand the mechanisms of DEPs by incorporating clinical CSF and plasma samples, as well as gene knockout mouse models.

## Conclusion

We first investigate the neuroprotective effects of MH on the hippocampus proteome of experimental models of cerebral IR injury. The general overview of protein regulation presented in our study provides insight into the potential mechanisms of neuroprotection of MH treatment. More importantly, we revealed four key DEPs (VCAM-1, S100A8, CaMKK2 and MKK7), which may guide future work and may have implications in clinical settings where MH is used to treat cerebral IR injury.

### Supplementary Information

Supplementary Figure S1.

Supplementary Figure S2.

Supplementary Table S1.

## Data Availability

The datasets used and/or analyzed during the current study are available from the corresponding author on reasonable request.
