# Supplementary material for: Proteome profiling of hippocampus reveals the neuroprotective effect of mild hypothermia on global cerebral ischemia–reperfusion injury in rats
Source: Sci Rep. 2023 Sep 2;13:14450. doi: 10.1038/s41598-023-41766-2 (PMC10475051; doi:10.1038/s41598-023-41766-2)
Supplement: Supplementary file 1 — Supplementary Figure S1. [file 41598_2023_41766_MOESM1_ESM.pdf]

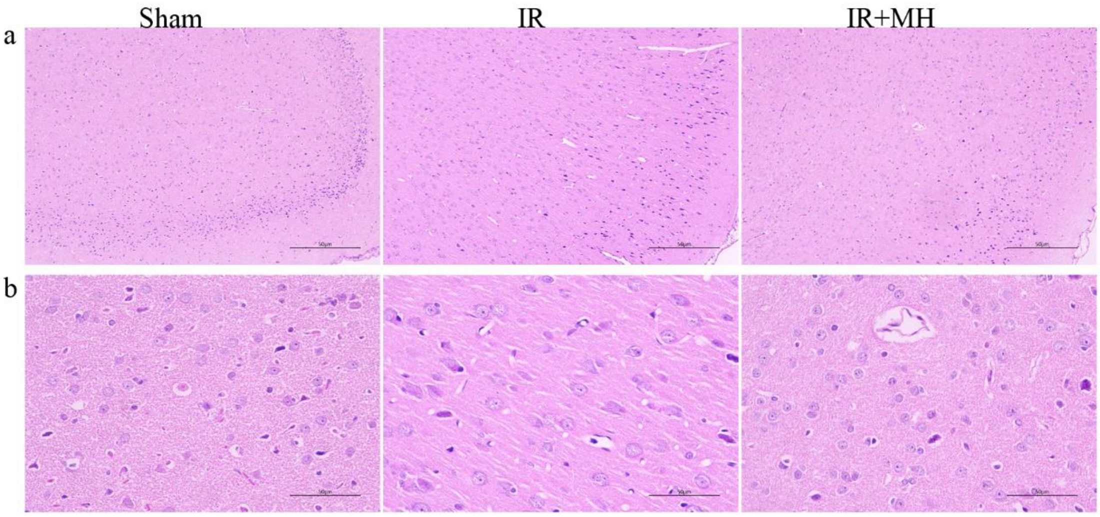

Figure S1. Protective effects of mild hypothermia on cerebral cortex neurological damage after cerebral ischemia-perfusion injury. (a: 10X, b: 40X)

As shown in Figure S1, HE staining revealed that the structure of cerebral cortex neurons in the sham group was complete, and the cell arrangement and morphology were normal. The IR group showed edema of neurons, gaps widen, and cellular structure is intact. In the IR+MH group, the morphology of nerve cells in cerebral cortex was basically improved.
