# Supplementary material for: Proteome profiling of hippocampus reveals the neuroprotective effect of mild hypothermia on global cerebral ischemia–reperfusion injury in rats
Source: Sci Rep. 2023 Sep 2;13:14450. doi: 10.1038/s41598-023-41766-2 (PMC10475051; doi:10.1038/s41598-023-41766-2)
Supplement: Supplementary file 2 — Supplementary Figure S2. [file 41598_2023_41766_MOESM2_ESM.pdf]

a. Western blotting analysis of VCAM-1 in the three groups

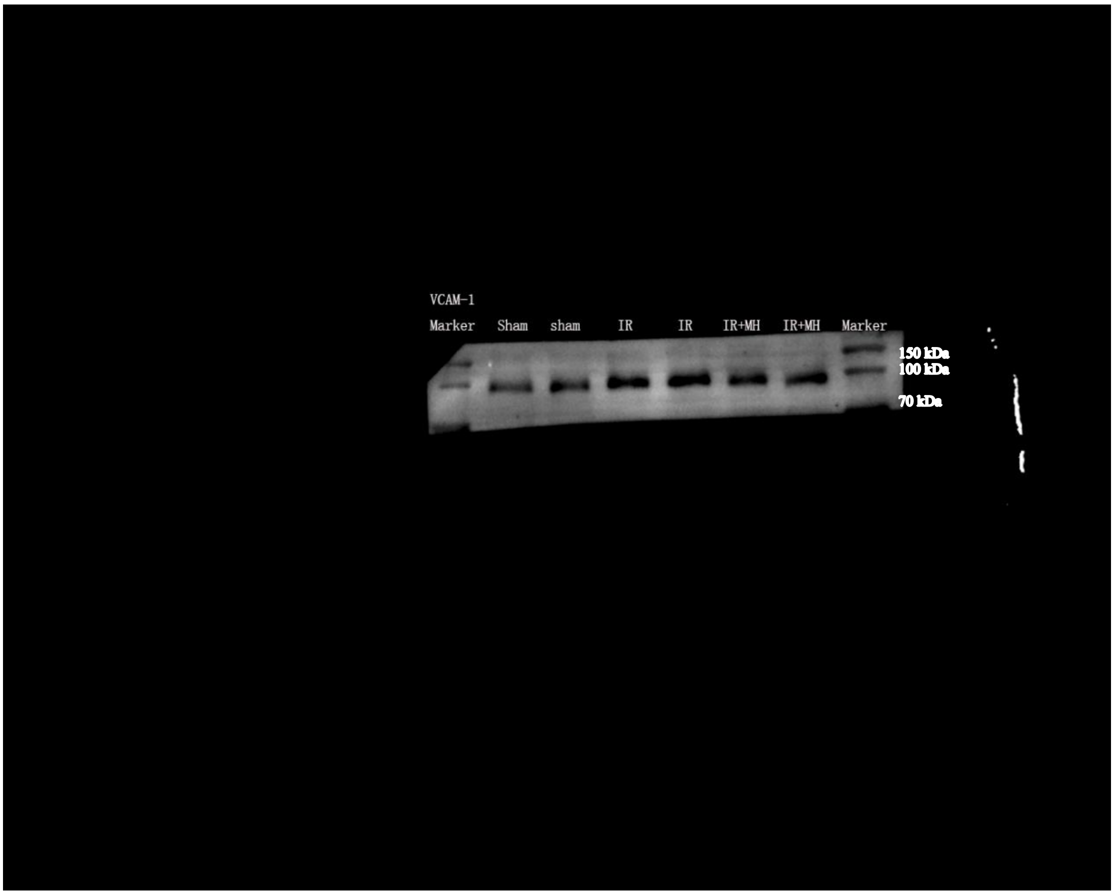

b. Western blotting analysis of VCAM-1 in the three groups.

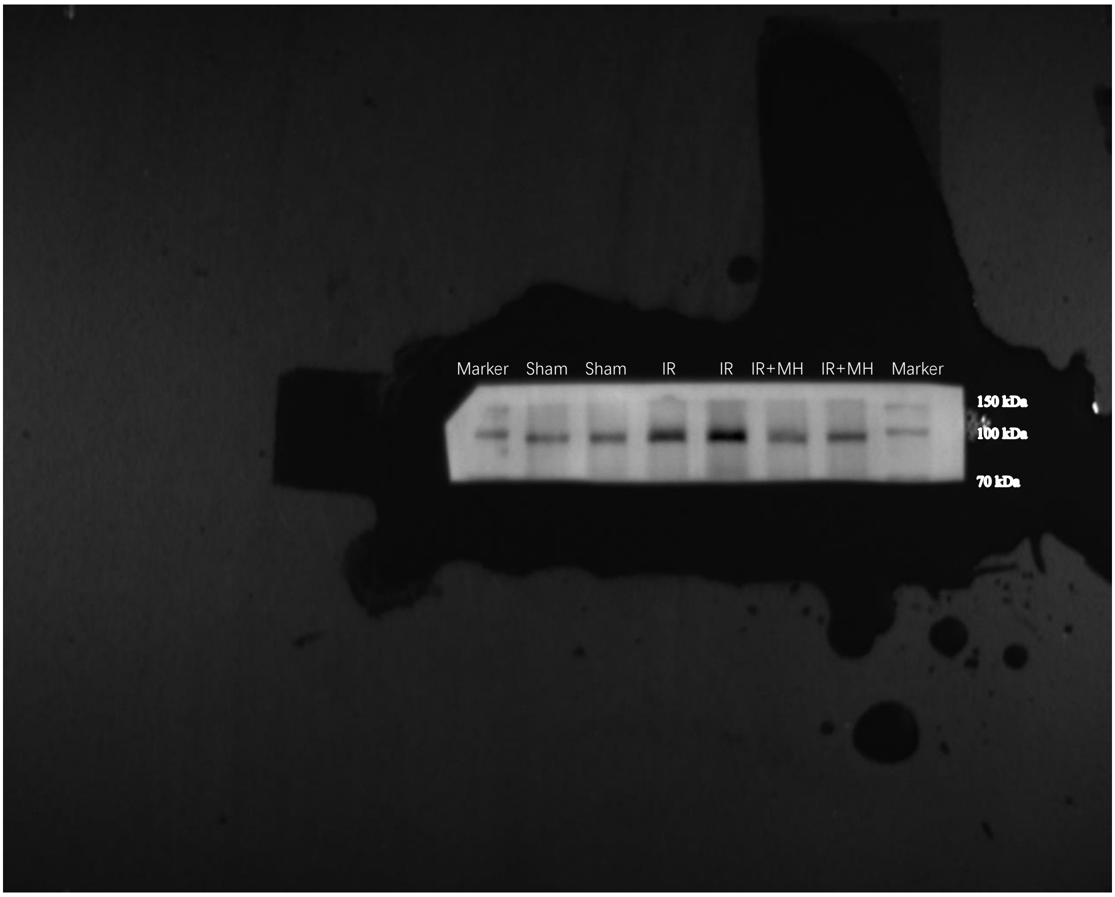

c. Western blotting analysis of CaMKK2 in the three groups.

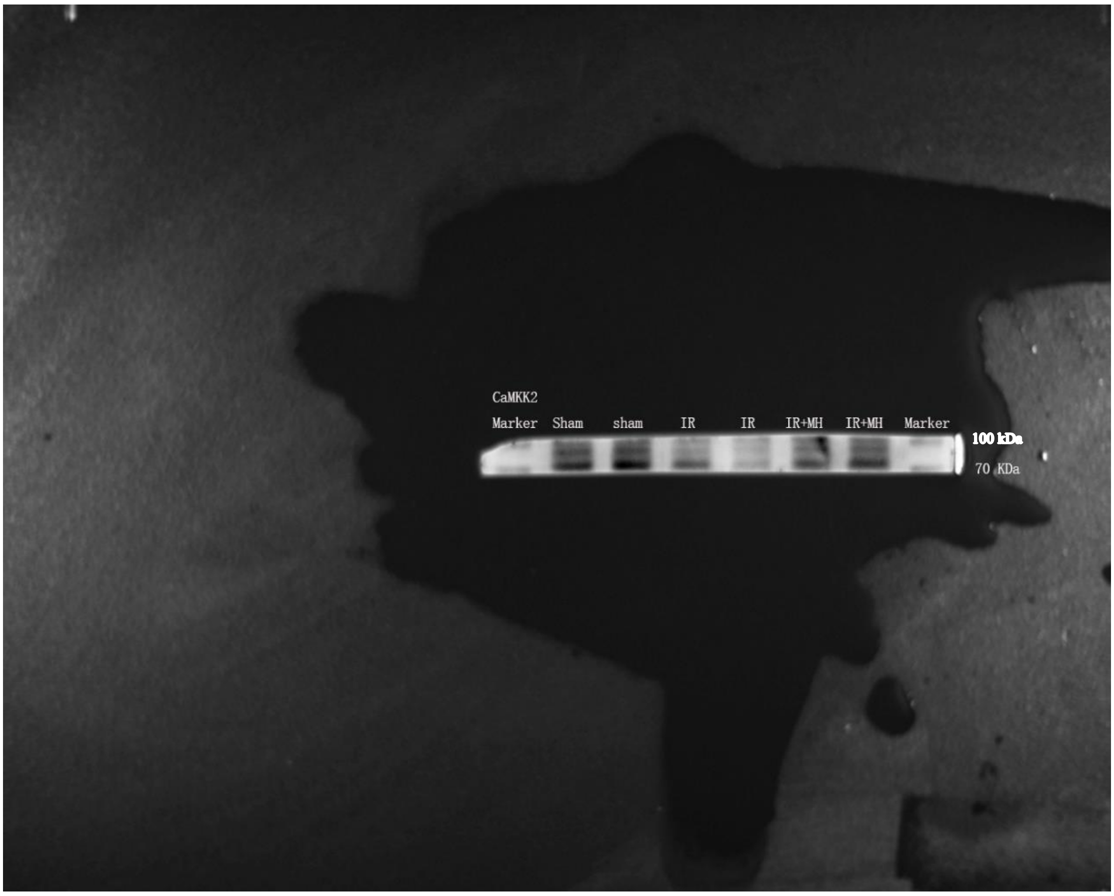

d. Western blotting analysis of CaMKK2 in the three groups.

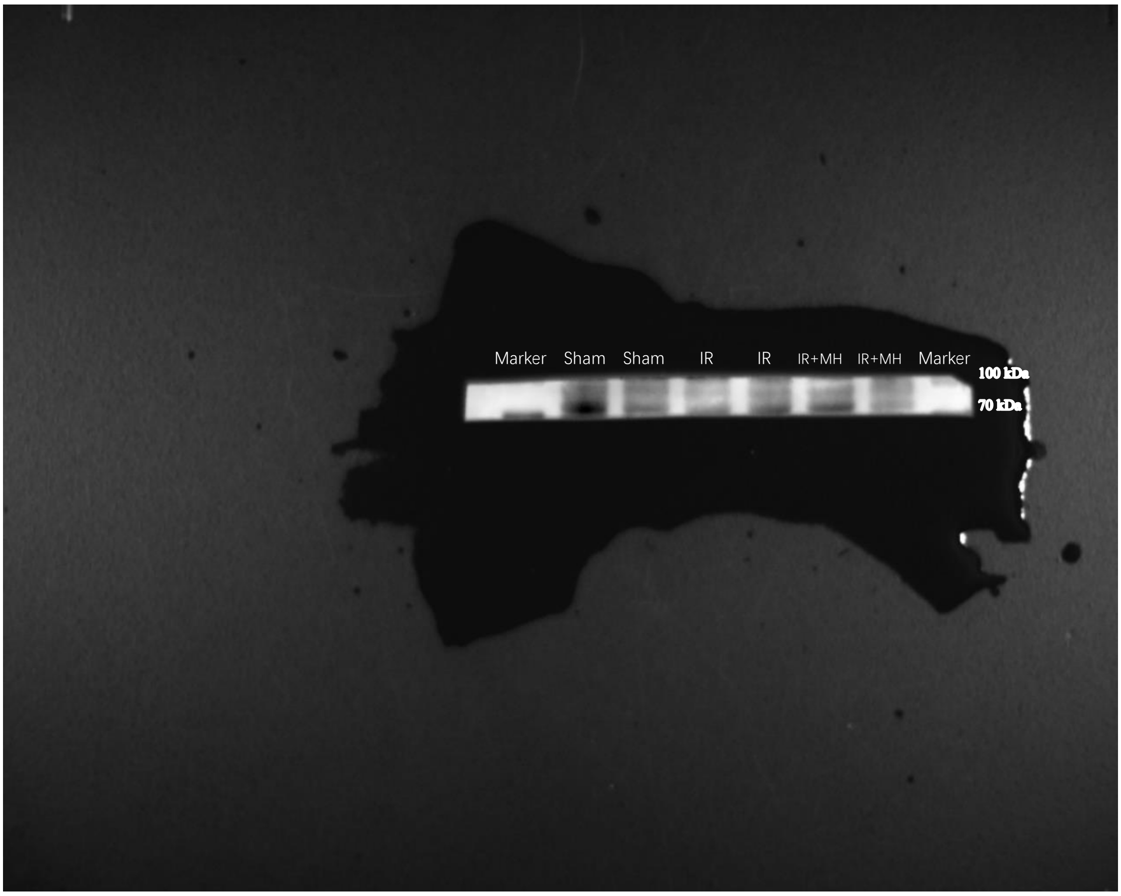

e. Western blotting analysis of MKK7 in the three groups.

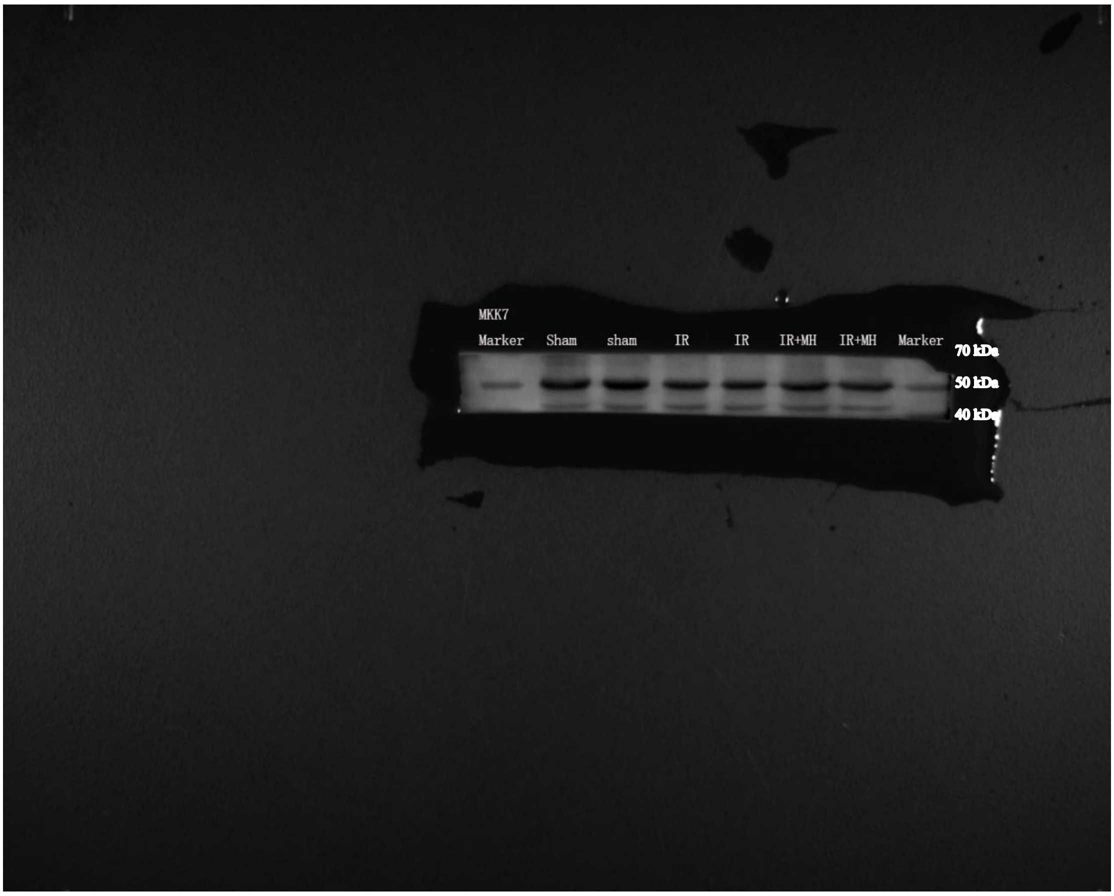

f. Western blotting analysis of MKK7 in the three groups.

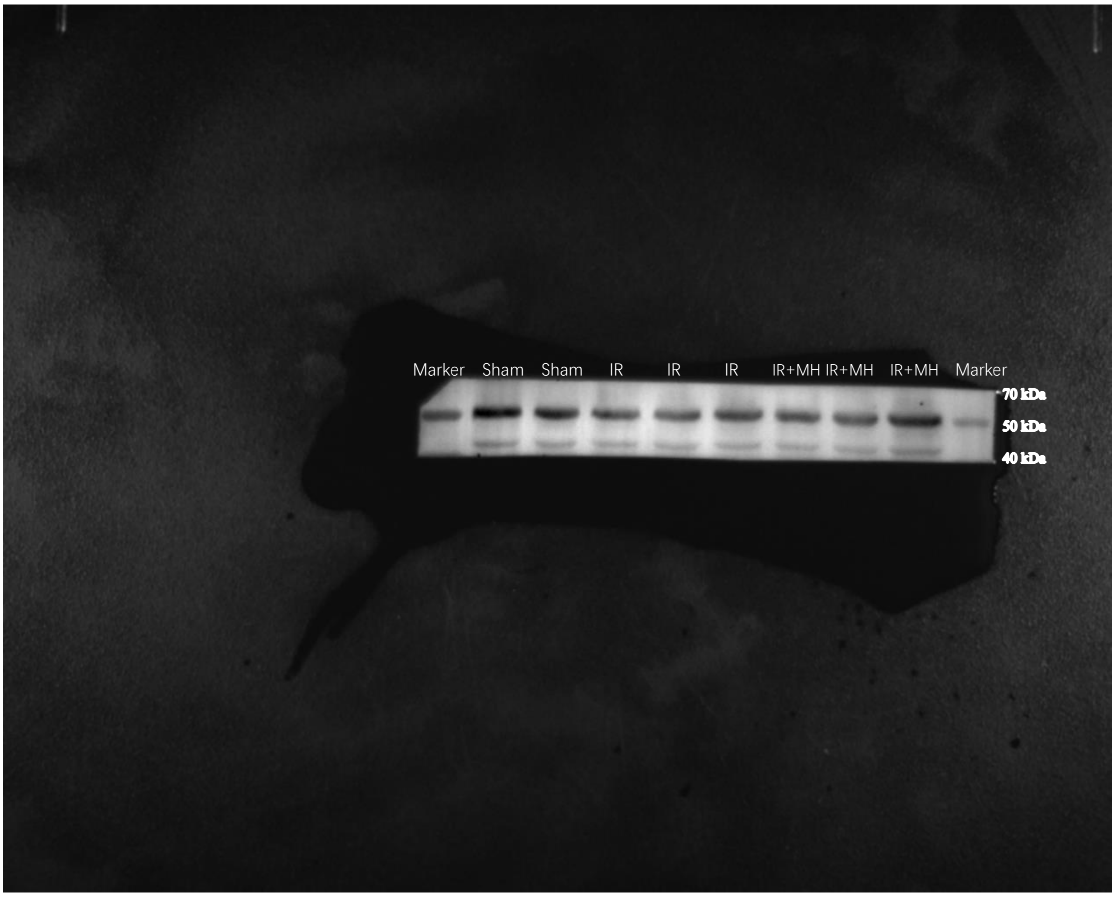

g. Western blotting analysis of S100A8 in the three groups.

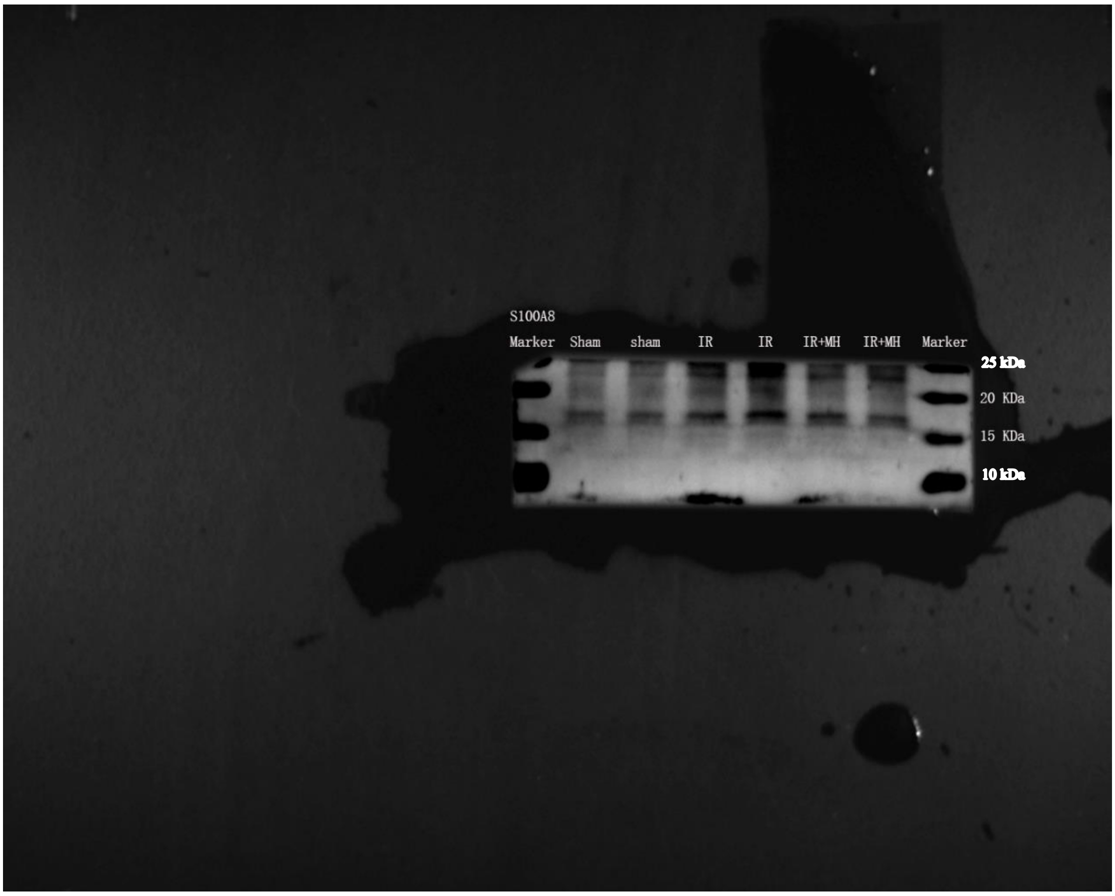

h. Western blotting analysis of S100A8 in the three groups.

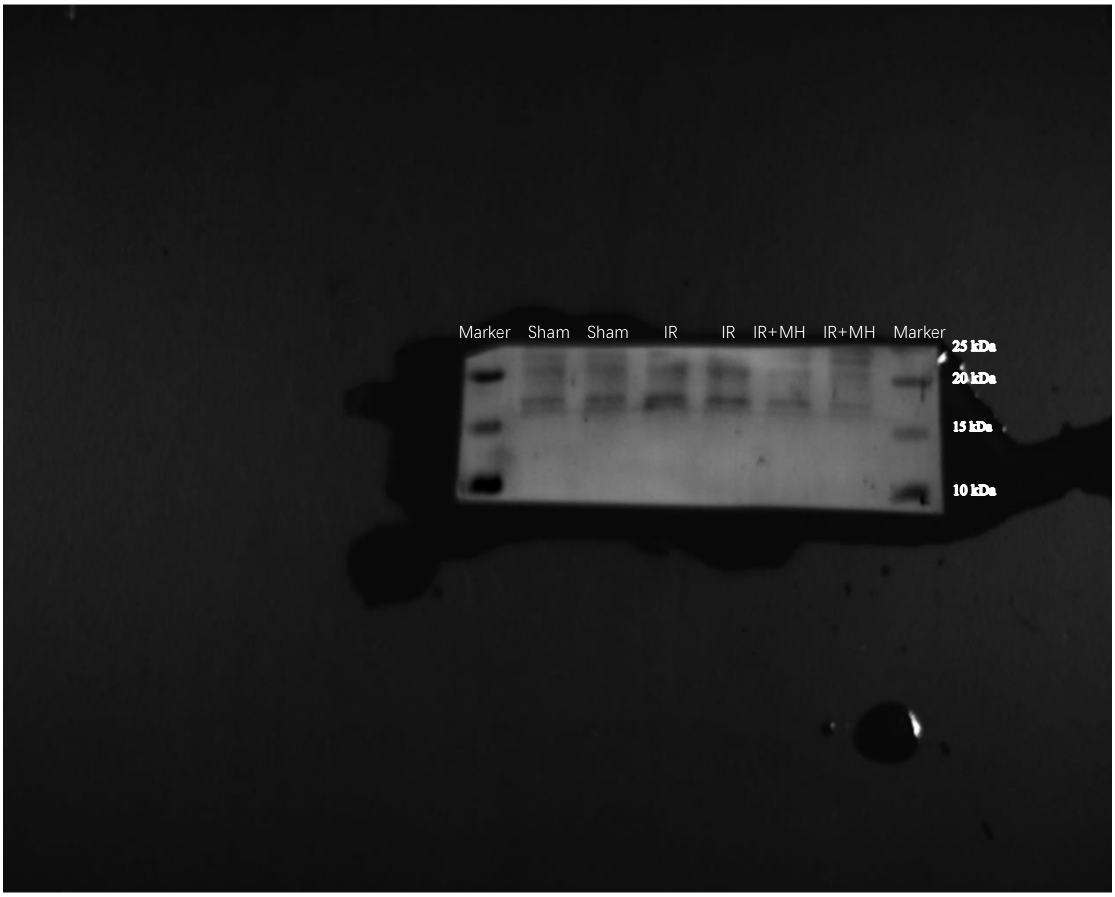

i. Western blotting analysis of GAPDH in the three groups.

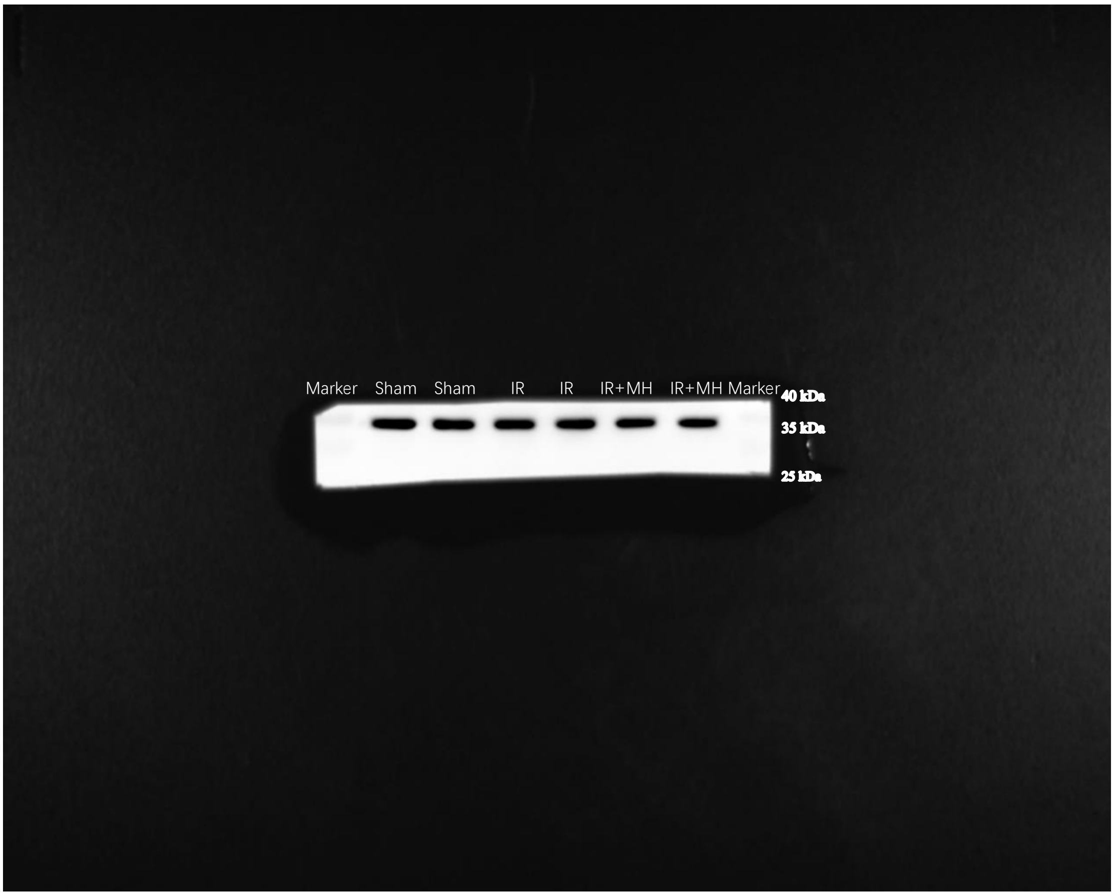

j. Western blotting analysis of GAPDH in the three groups.

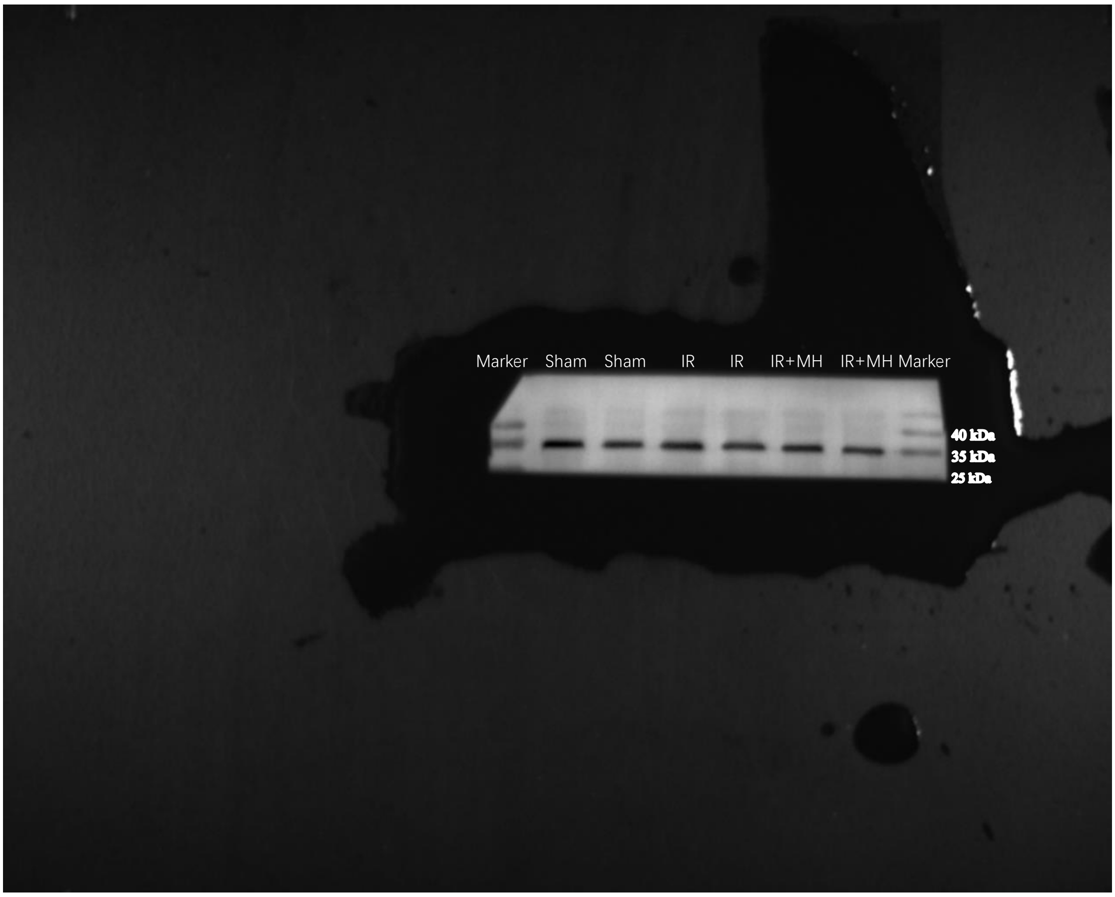

k. Western blotting analysis of GAPDH in the three groups.

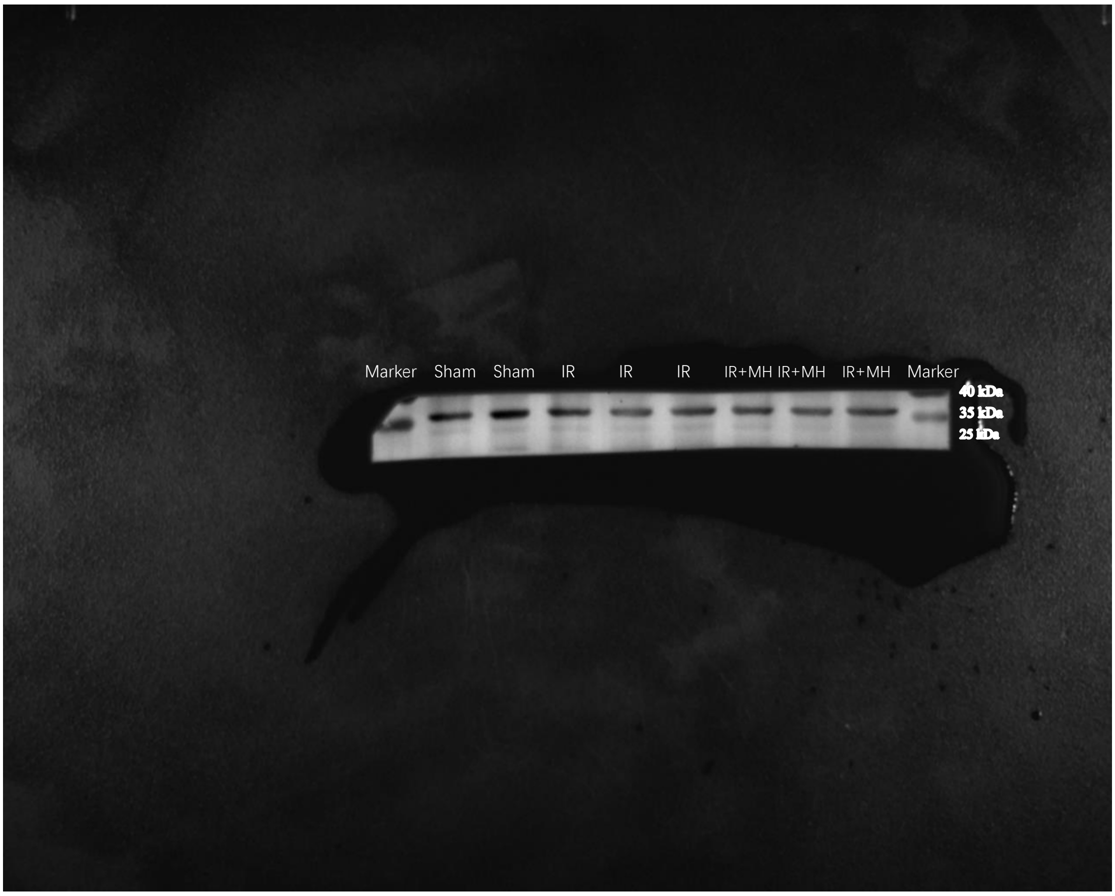

Sham: Sham-operation group; IR: cerebral IR injury followed by normothermia (37°C) group; IR+MH: cerebral IR injury followed by 4 h of MH (32°C) group.
